# Juvenile Myoclonic Epilepsy Shows Potential Structural White Matter Abnormalities: A TBSS Study

**DOI:** 10.3389/fneur.2018.00509

**Published:** 2018-06-29

**Authors:** Martin Domin, Sabine Bartels, Julia Geithner, Zhong I. Wang, Uwe Runge, Matthias Grothe, Soenke Langner, Felix von Podewils

**Affiliations:** ^1^Functional Imaging Unit, Department of Diagnostic Radiology and Neuroradiology, University Medicine Greifswald, Greifswald, Germany; ^2^Department of Neurology, Epilepsy Center, University Medicine Greifswald, Greifswald, Germany; ^3^Epilepsy Center Berlin-Brandenburg, Ev. Krankenhaus Königin Elisabeth Herzberge, Berlin, Germany; ^4^Epilepsy Center, Neurological Institute, Cleveland Clinic Foundation, Cleveland, OH, United States; ^5^Diagnostic and Interventional Neuroradiology, University Medicine Rostock, Rostock, Germany

**Keywords:** juvenile myoclonic epilepsy, photoparoxysmal responses, microstructural abnormalities, tract-based spatial statistics, network dysfunction

## Abstract

**Background:** Several studies on patients with juvenile myoclonic epilepsy (JME) showed widespread white matter (WM) abnormalities in the brain. The aim of this study was to investigate potential structural abnormalities in JME patients (1) compared to healthy controls, (2) among JME subgroups with or without photoparoxysmal responses (PPR), and (3) in correlation with clinical variables.

**Methods:** A selection of 31 patients with JME (12 PPR positive) and 27 age and gender matched healthy controls (HC) were studied at a tertiary epilepsy center. Fractional anisotropy (FA) was calculated and intergroup differences analyzed using Tract Based Spatial Statistics (TBSS).

**Results:** Compared to HC the JME group showed reduced FA widespread and bilateral in the longitudinal fasciculus, inferior fronto-occipital fasciculus, corticospinal tract, anterior and posterior thalamic radiation, corona radiata, corpus callosum, cingulate gyrus and external capsule (*p* < 0.01). Subgroup analysis revealed no significant differences of WM alterations between PPR positive and negative patients and with clinical and epilepsy-related factors.

**Conclusions:** Widespread microstructural abnormalities among patients with JME have been identified.Prior findings of frontal and thalamofrontal microstructural abnormalities have been confirmed. Additionally, microstructural abnormalities were found in widespread extra-frontal regions that may help to validate pathophysiological concepts of JME.

## Introduction

Juvenile myoclonic epilepsy (JME, Janz syndrome) is a genetic generalized epilepsy (GGE) syndrome with a prevalence of 5–11% among all epilepsies (1, 2). The occurrence of bilateral myoclonic seizures (BMS) is mandatory for the diagnosis of JME, often combined with absence seizures (ABS) and/or generalized tonic-clonic seizures (GTCS) (1). The interictal electroencephalography (EEG) characteristically shows generalized spikes and poly-spike waves ≥3 Hz (1, 3). About 30% of patients with JME show photoparoxysmal responses (PPR), defined as the occurrence of spikes, poly-spike-waves or repetitive spikes in the EEG in response to intermittent photic stimulation (PS) (4, 5).

Diffusion tensor imaging (DTI) is a non-invasive magnetic resonance imaging (MRI) based structural imaging modality, that enables the depiction and quantification of white matter (WM) fiber tracts of the brain *in vivo*. Important DTI parameters are fractional anisotropy (FA) and mean diffusivity (MD) (6, 7). Compared to region-based approaches tract-based spatial statistics (TBSS) is a relatively new modality using whole brain DTI to generate a pseudo-anatomical “skeleton” of WM tracts on the basis of FA images (3, 8, 9).

Prior studies among patients with JME using DTI consistently found thalamocortical and cortico-cortical network abnormalities (2, 9–12). Furthermore, microstructural alterations in the genu of the internal capsule, the ascending reticular activating system (ARAS), and the ventromedial thalamus (VMT) were reported in the subgroup of PPR positive JME patients (2). A very recent TBSS study showed altered WM connectivity in the left corpus callosum (CC), thalamic radiation, superior longitudinal fasciculus (SLF), and corticospinal tract (CST), presuming an association with frontal cognitive and motor dysfunction in JME patients (13).

The aim of this study was to identify microstructural abnormalities in JME compared to healthy controls and between the subgroups of PPR positive (pPPR) and negative (nPPR) JME patients using TBSS. Furthermore, our aim was to determine potential associations between certain structural abnormalities and clinical features typical for JME.

## Materials and methods

### Patients

This study was approved by the local Institutional Review Board of the University Medicine Greifswald and conducted at a tertiary care epilepsy center (total population of the catchment area ≈500,000). All subjects gave written informed consent in accordance with the Declaration of Helsinki.

Thirty-one patients with JME and 28 healthy controls (HC) were prospectively recruited from the inpatient and outpatient clinic.

Inclusion criteria were as follows: (1) diagnosis of JME, (2) normal neurological examination and overall intelligence, (3) at least one abnormal routine EEG showing generalized spikes and/or poly-spike-waves. Cases with a history of epilepsy syndromes other than JME and severe brain trauma were excluded. Healthy controls had a normal clinical MRI and routine EEG examinations, no history of neurological disease and brain trauma, and no family history of epilepsy. Diagnosis of JME was made on the basis of the patients' medical history and EEG. Patients were considered as PPR positive (pPPR) if epileptiform discharges only occurred in response to intermittent PS; PPRs were classified according to classification scheme by Waltz et al.(14) Clinical data were collected by reviewing the medical records and during an interview.

### Data acquisition

MRI was conducted with a 3-Tesla MRI-Scanner (Verio, Siemens, Erlangen, Germany) using a 32-channel head coil. We applied a Siemens MDDW (Multi Directional Diffusion Weighting) sequence with the following parameter setup: voxel size isotropic 1.8 mm^3^, matrix size 128 × 128 voxel, 80 slices, 1 acquisition and 64 directions. TR was 15,300 ms, TE: 107 ms and the total scan time was 17 min.

#### Preprocessing

The measured raw DICOM data was converted into NIFTI format using *dcm2nii*, which is part of the neuroimaging tool MRIcron. The tool *eddy_correct*, part of FSL, Smith et al. (15) was used to correct the diffusion-weighted data with respect to subject motion and deformations introduced by eddy current artifacts of the MRI scanner. Fractional anisotropy (FA) images were created by fitting a tensor model to the raw diffusion data using FSL DTI-FIT.

#### Tract-Based Spatial Statistics (TBSS)

Preparation for voxelwise statistical analysis of the FA data was carried out using the TBSS approach of FSL [Tract-Based Spatial Statistics, (8)]. We chose to replace the FLIRT/FNIRT registration of FSL with the tensor-based registration approach of DTI-TK (16), as Bach and colleagues (17) were showing this to be preferable over the default FSL approach. Here, the registration is based on the whole tensor matrix in each voxel, whereas the FSL mechanism utilizes the scalar FA values only. We followed the procedure and the manual provided by Zhang and colleagues (http://dti-tk.sourceforge.net/), where a first, crude group-wise template is created, which is iteratively refined by using affine and non-linear registrations, incorporating the tensor matrices instead of FA values. This process included the calculation of subject-specific non-linear transformations into a common template space (IIT; Illinois Institute of Technology) and a subsequent normalization of the individual FA images. These were then combined and thinned using a projection technique to create an average FA skeleton consisting of locally maximal FA values. Finally, each subject's aligned FA data was projected onto this skeleton and the resulting data fed into voxel-wise cross-subject statistics.

Because of the spatial differences of the IIT template and the MNI-ICBM 152 nonlinear 6th generation, even if they are supposed to be in the same MNI template space, a non-linear registration between these two templates had to be calculated, as most atlases or regions-of-interest reside in the MNI-ICBM 152 space.

### Statistics

FSL's tool for nonparametric permutation inference on neuroimaging data, *randomize* (18), was used to carry out the voxel-wise cross-subject statistics. This approach belongs to the permutation or randomization methods, which are used when the null distribution of the data is not known. This can be the case, if e.g., the noise of the data does not follow a simple distribution, as can be found in MRI data that contains noise following a Rician distribution. Voxel-wise statistics were corrected for multiple comparisons using the Family-wise-error approach (FWE) and, if necessary, contrast-specific *p*-values were corrected for the number of contrasts per test. Permutation-based testing included *t*-tests for group comparisons as well as for correlations with clinical data, which is permutationally equivalent to a partial correlation. We used 50,000 permutations (FSL default = 5,000) per test, as this number significantly reduces uncertainties of the *p*-values. Threshold-free cluster enhancement (TFCE) was used to enhance cluster-like structures without the need of preset clustering thresholds (TBSS-specific randomize parameter –T2).

At last, the FSL tool atlasquery automatically compared statistically significant voxels with common white matter atlases provided by Mori and colleagues (ICBM-DTI-81 white-matter labels atlas, JHU white-matter tractography atlas), which are part of the FSL software package (19).

## Results

Relevant clinical data of all patients and HC included in the study are given in Table 1. Thirty-one patients with JME (23 female) were enrolled (mean age 31.7 years; SD ± 10.95, range 18–62); mean age at epilepsy onset was 15.9 years (SD ± 6.4, range 2–36) and mean duration of epilepsy 15 years (SD ± 9.9, range 2–41). Twelve (38.7%) were pPPR [each classified as PPR type III or IV according to Waltz et al. (14)]. Seizure-free rate was 39% (*n* = 12), all of these patients were treated with AED (see Table 1).

**Table 1:** Clinical data of all patients and controls included in the study.

	**Patients**	**Controls**
*N* =	31	28
Female (%)	23 (74%)	15 (54%)
Age: mean (range); SD	31.7 years (18–62); ± 10.9	27 years (19–34); ± 4.6
EO: mean (range); SD	15.9 years (2–36); ± 6.4	
ED: mean (range); SD	15 years (2–41); ± 9.9	
Seizure type: only BMS	4 (13%)	
+ GTCS	10 (32%)	
+ ABS	3 (10%)	
+ ABS + GTCS	12 (39%)	
unknown	2 (7%)	
PPR	12 (39%)	
SF [with/without AED]	12 (39%) [12/0]	
NSF [with/without AED]	19 (61%) [16/3]	

*ABS, absence seizures; AED, antiepileptic drug; BMS, bilateral myoclonic seizures; ED, epilepsy duration; EO, epilepsy onset; GTCS, generalized tonic-clonic seizures; NSF, non seizure-free; PPR, photoparoxysmal response; SD, standard deviation; SF, seizure-free*.

### JME vs. healthy controls

**Figure 1:**
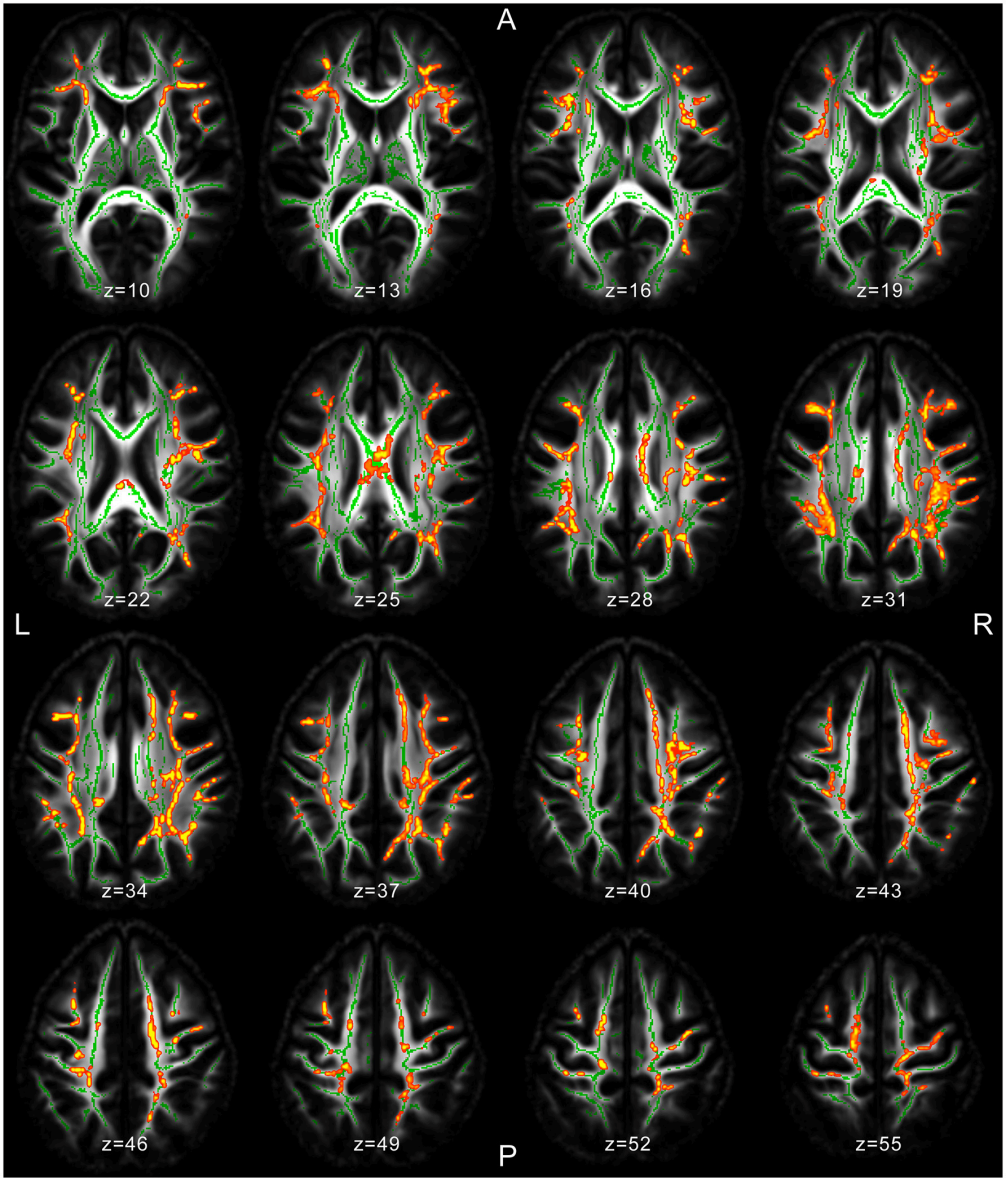
MRI images of significant TBSS clusters in patients with JME compared to healthy controls superimposed to the skeleton (light green).

Several significant microstructural abnormalities among patients with JME compared to HC have been found (Table 2; Table S3 for cluster-related statistical information; Figure 1). Analysis of regional maxima revealed most significant FA reduction in the following bilateral regions: (1) superior and inferior longitudinal fasciculus (SLF/ILF), inferior fronto-occipital fasciculus (IFOF), anterior and posterior thalamic radiation (ATR/PTR), anterior, superior, and posterior corona radiata (ACR/SCR/PCR), body and splenium of corpus callosum (CC), cingulate gyrus (CG), hippocampus, corticospinal tract (CST), Forceps major (FMa) and minor (FMi), uncinate fasciculus and external capsule (*p* < 0.01). Considering a conservative threshold of *p* < 0.01 significant results were also found only on the right side in the anterior and posterior limb of internal capsule.

**Table 2:** TBSS results–JME vs. healthy controls.

**JHU_White-Matter_Tractography_Atlas**	**Average probability**	**Significance**
Anterior thalamic radiation L	1.11	0.01
Anterior thalamic radiation R	0.87	0.01
Cingulum (cingulate gyrus) L	0.20	0.01
Cingulum (cingulate gyrus) R	0.13	0.01
Cingulum (hippocampus) L	0.02	0.01
Cingulum (hippocampus) R	0.01	0.01
Corticospinal tract L	0.78	0.01
Corticospinal tract R	0.96	0.01
Forceps major	0.38	0.01
Forceps minor	2.54	0.01
Inferior fronto-occipital fasciculus L	1.42	0.01
Inferior fronto-occipital fasciculus R	1.11	0.01
Inferior longitudinal fasciculus L	1.06	0.01
Inferior longitudinal fasciculus R	0.73	0.01
Superior longitudinal fasciculus (temporal part) L	0.98	0.01
Superior longitudinal fasciculus (temporal part) R	0.69	0.01
Superior longitudinal fasciculus L	2.35	0.01
Superior longitudinal fasciculus R	2.31	0.01
Uncinate fasciculus L	0.50	0.01
Uncinate fasciculus R	0.28	0.01
**JHU_ICBM-DTI-81_White-Matter_Labels**	**Overlap percentage**	**Significance**
Body of corpus callosum	4.29	0.01
Splenium of corpus callosum	0.08	0.01
Anterior limb of internal capsule R	0.11	0.01
Posterior limb of internal capsule R	0.02	0.01
Anterior corona radiata R	1.67	0.01
Anterior corona radiata L	2.03	0.01
Superior corona radiata R	3.33	0.01
Superior corona radiata L	0.21	0.01
Posterior corona radiata R	1.18	0.01
Posterior corona radiata L	0.31	0.01
Posterior thalamic radiation (include optic radiation) R	0.27	0.01
Posterior thalamic radiation (include optic radiation) L	0.01	0.01
External capsule R	0.06	0.01
External capsule L	0.03	0.01
Superior longitudinal fasciculus R	6.91	0.01
Superior longitudinal fasciculus L	5.66	0.01

*L, left hemisphere; R, right hemisphere*.

### Photoparoxysmal responses: pPPR vs. nPPR

Inter-group comparison between pPPR and nPPR JME revealed no significant FA differences. See Tables S4–S7 for raw uncorrected statistical results.

### Correlation with epilepsy duration

No significant correlation was found between FA and duration of epilepsy (corrected for age and sex; Table 1). see Tables S8, S9 for raw uncorrected statistical results.

## Discussion

The aim of this study was to identify potential structural WM abnormalities in JME compared to HC and in between the two JME subgroups of pPPR and nPPR cases using TBSS. We additionally investigated clinical and epilepsy-related factors for an association with WM alterations. This TBSS analysis identified widespread FA reductions among patients with JME.

A very recent TBSS study among JME patients found microstructural abnormalities in the CC, thalamic radiation, SLF, and CST (13). This is consistent with both several of our findings in JME and prior findings among GGE patients in general (2, 9, 11) and underlines the reproducibility in identifying abnormal diffusion metrics in GGE patients using TBSS. Compared to HC our study reveals significant FA reductions in patients with JME bilateral in the previously described WM regions (SLF, thalamic radiation, CC, and CST), as well as the IFOF, FMa, FMi, CG, hippocampus, and—in addition to previously described regions—bilateral in the corona radiata.

FA decrease reflects an impaired microstructural integrity likely reflecting reduced myelination of WM tracts, which was found to play a pivotal role in the pathophysiology of generalized epilepsies (2, 9, 11, 12). In addition to alterations of the thalamo-cortical network and the CC among JME patients, histopathological findings of “microdygenesis” predominantly in the frontal lobe, including the frontal CC, support the hypothesis of a parent pathophysiological role of thalamo-frontal as well as inter-frontal networks in generalized epilepsies and challenge the existing hypotheses of the thalamus as a key structure in generalized epilepsies.

In the past, the focus of region of interest (ROI) seed-based approaches was mainly on frontal areas and the thalamus (11, 20). More recent studies using whole brain approaches (TBSS) found network alterations also beyond the frontal lobes, including more posterior regions and particularly the precuneus (9, 21, 22), which is known to play a role as a part of the functional “default mode” network, in cognitive functions (23), and in generalized spike-wave discharges in GGE (22, 24, 25). Strong interconnections between the precuneus and both, parieto-occipital primary-visual and frontal areas are known (26, 27). Our TBSS findings indicate an involvement of large parts of the WM, predominantly comprising structures of the bilateral longitudinal fasciculus (SLF), the cortico-thalamic (corona radiata), and cortico-spinal (CST) connections, which are known to be linked with the precuneus. Conclusively, in addition to the previously described relation of widespread microstructural abnormalities and cognitive and personality characteristics in JME patients (28), our findings may lead to the hypothesis that the precuneus is part a widespread network and thereby involved in both cognitive characteristics and generalized spike-wave discharges in JME patients.

The CST contains motor fibers predominantly originating in the motor cortex targeting spinal alpha motor neurons, and the corona radiata (anterior/superior part) those between the thalamus and premotor cortex, including the supplementary motor area (SMA) (29). BMS and GTCS are predominantly characterized by motor symptoms. Therefore, it can be postulated that microstructural alterations of cortico-spinal and thalamo-frontal connections are an essential component of the propagation network of generalized seizures as previously suggested (2, 3, 9, 12, 13) and may even reflect an increased epileptogenicity with a lower threshold to generate generalized seizures, notably those with predominant motor symptoms. Taken together, an epileptogenic network involving cortico-cortical, thalamo-cortical, and cortico-spinal connections in patients with JME can be assumed. Different components of this network may be of special significance for certain clinical characteristics of JME, such as circadian rhythm of seizures (thalamus) and seizures with predominantly motor symptoms (frontal cortex, thalamo-frontal connections, CST, ACR/SCR, internal capsule). It can be hypothesized that the extent of microstructural abnormalities within this network may determine the clinical subtype of JME in the individual patient, however, this was not examined by our study protocol and should be a goal for future studies.

### Photoparoxysmal responses (PPR)

Photosensitive JME is considered to be a subtype of JME with a higher seizure risk (30). Additionally, increased FA has been shown in a cohort of pPPR JME patients in the ARAS and the VMT (2). Both ARAS and thalamus are known to be crucial in the circadian rhythm regulation (31), which may trigger the generation of seizures and may also be one explanation for the association of seizures with awakening (2). Furthermore, networks involving retinal ganglion cells, ARAS, lateral geniculate nucleus, and the primary-visual cortex were shown to be essential in the epileptologenesis of JME and may also explain the association with awakening (2). Nevertheless, albeit microstructural WM abnormalities were found in the total JME group, our study revealed no significant differences between those who were pPPR and nPPR (see Tables S4–S7 for raw uncorrected statistical results).

Several limitations of our study need to be considered. Due to the relatively small group of 31 patients the possibility of Type-2 statistical errors should be considered. Nevertheless, the single-center approach ensures a consistent syndrome characterization of the included patients and increases the internal validity of the data. Additionally, although JME is considered an easy-to-treat epilepsy syndrome, only 39% (*n* = 12) of our patients were seizure-free. Assuming favorable outcome as a reason for renunciation from our epilepsy center, a potential selection toward more intractable patients has to be considered.

Despite the above limitations, our results amend the knowledge on WM abnormalities in JME. Microstructural variations of JME patients support the hypothesis that JME is a network dysfunction involving both widespread cortical and subcortical (thalamus, thalamo-cortical connections) structures. Within this network the precuneus may play a pivotal role in the connection of cortico-cortical (fronto-occipital) and cortico-subcortical (thalamus) structures. Compared to whole brain approaches region-based approaches may not be sufficient to identify the spatial extent of microstructural WM abnormalities in JME. Taken together, our findings may help to generate hypotheses about structural and network connectivity and contribute to better understand the pathophysiology and epileptic networks in JME.

## Disclosure

MG reports travel reimbursement from Novartis Pharma, Teva Pharma, and BiogenIdec and research grants from the Federal Ministry for Research and Education in Germany. UR received personal compensation for consulting services from UCB Pharma, Eisai, and Desitin and research grants from UCB Pharma. FvP obtained honoraria for speaking engagements from Desitin, Eisai, Bial, and UCB Pharma, and was part of speaker's bureau of Desitin, Eisai, Bial, and UCB Pharma. The other authors have nothing to disclose. This research did not receive any specific grant from funding agencies in the public, commercial, or not-for-profit sectors.

## Ethics statement

We confirm that we have read the Frontiers in Neurology position on ethics and procedures and confirm that this report is consistent with these guidelines.

## Author contributions

MD, FvP, and UR generated the research idea, study design, and concept. SB, FvP, JG, and MG acquired and analyzed the data and drafted the work. MD and SL analyzed the data. All authors made critical revisions for important intellectual content and interpreted the data. MD, FvP, and SB wrote the manuscript. All authors contributed to manuscript revision, read and approved the submitted version.

### Conflict of interest statement

The authors declare that the research was conducted in the absence of any commercial or financial relationships that could be construed as a potential conflict of interest.

## Abbreviations

ACR: anterior corona radiata
ARAS: ascending reticular activating system
ATR: anterior thalamic radiation
BMS: bilateral myoclonic seizures
CC: corpus callosum
CG: cingulate gyrus
CST: corticospinal tract
DTI: diffusion tensor imaging
EEG: electroencephalography
FA: fractional anisotropy
FMa: Forceps major
FMi: Forceps minor
GGE: genetic generalized epilepsy
GTCS: generalized tonic-clonic seizures
HC: healthy controls
IFOF: inferior fronto-occipital fasciculus
ILF: inferior longitudinal fasciculus
JME: juvenile myoclonic epilepsy
MD: mean diffusivity
MRI: magnetic resonance imaging
nPPR: PPR negative
PCR: posterior corona radiata
pPPR: PPR positive
PPR: photoparoxysmal responses
PS: photic stimulation
PTR: posterior thalamic radiation
ROI: region of interest
SCR: superior corona radiata
SD: standard deviation
SLF: superior longitudinal fasciculus
SMA: supplementary motor area
TBSS: tract based spatial statistics
VMT: ventromedial thalamus
WM: white matter.
