# Supplementary material for: Juvenile Myoclonic Epilepsy Shows Potential Structural White Matter Abnormalities: A TBSS Study
Source: Front Neurol. 2018 Jun 29;9:509. doi: 10.3389/fneur.2018.00509 (PMC6033991; doi:10.3389/fneur.2018.00509)
Supplement: Supplementary file 1 [file Data_Sheet_1.docx]

Supplementary Material

Juvenile myoclonic epilepsy shows potential structural white matter abnormalities: a TBSS study

Martin Domin, Sabine Bartels, Julia Geithner, Zhong Irene Wang, Uwe Runge, Matthias Grothe*, Soenke Langner, Felix von Podewils

*** Correspondence:** Corresponding Author: matthias.grothe@uni-greifswald.de

# Supplementary Tables

**Table 3** Significant clusters, their peak p-value and MNI coordinates of TBSS results (Patients<Healthy subjects, p<0.05 TFCE and FWE corrected). For the sake of brevity only the 25 largest clusters are shown.

| **Cluster size** | **p-value peak** | **X (mm)** | **Y (mm)** | **Z (mm)** |
| --- | --- | --- | --- | --- |
| 4142 | 0,003 | -28 | -52 | 31 |
| 4089 | 0,002 | 29 | -31 | 38 |
| 1476 | 0,005 | 19 | 11 | 39 |
| 1069 | 0,003 | 22 | -56 | 30 |
| 923 | 0,008 | -36 | 27 | 11 |
| 798 | 0,005 | 2 | -7 | 24 |
| 621 | 0,023 | -34 | 0 | -29 |
| 594 | 0,031 | -11 | -21 | -24 |
| 537 | 0,024 | -39 | -27 | -5 |
| 513 | 0,008 | 39 | 29 | 10 |
| 445 | 0,032 | 47 | -23 | -18 |
| 441 | 0,033 | -31 | -66 | 13 |
| 401 | 0,014 | -12 | 27 | 13 |
| 400 | 0,009 | -10 | -6 | 59 |
| 398 | 0,014 | -28 | -10 | 20 |
| 395 | 0,006 | 29 | -13 | 17 |
| 371 | 0,037 | 11 | -13 | 15 |
| 370 | 0,032 | 40 | -20 | -9 |
| 369 | 0,013 | -17 | 46 | 19 |
| 329 | 0,013 | -34 | -68 | 26 |
| 322 | 0,014 | -12 | 54 | -13 |
| 320 | 0,026 | 22 | -15 | -5 |
| 289 | 0,013 | -23 | -67 | 26 |
| 280 | 0,008 | 44 | 23 | 14 |
| 269 | 0,019 | 42 | -39 | 20 |

**
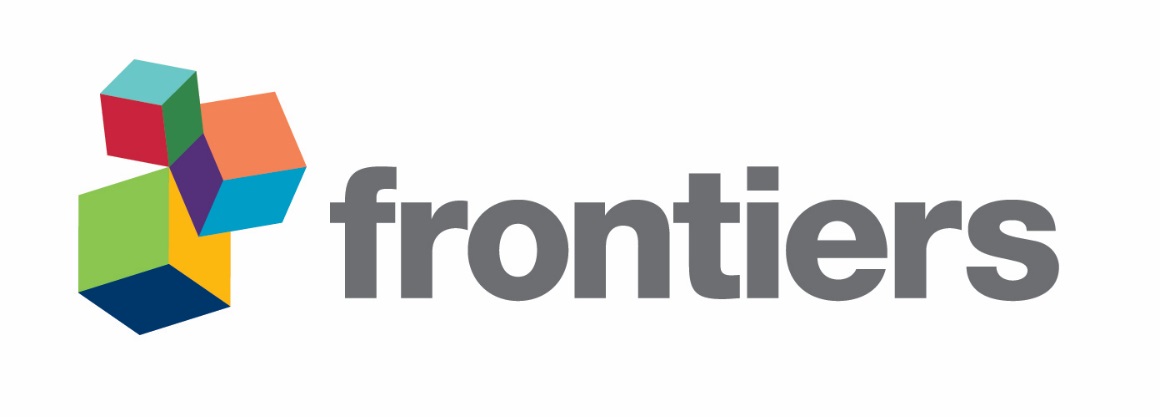
**
