# Supplementary material for: Juvenile Myoclonic Epilepsy Shows Potential Structural White Matter Abnormalities: A TBSS Study
Source: Front Neurol. 2018 Jun 29;9:509. doi: 10.3389/fneur.2018.00509 (PMC6033991; doi:10.3389/fneur.2018.00509)
Supplement: Supplementary file 2 [file Data_Sheet_2.docx]

Supplementary Material

Juvenile myoclonic epilepsy shows potential structural white matter abnormalities: a TBSS study

Martin Domin, Sabine Bartels, Julia Geithner, Zhong Irene Wang, Uwe Runge, Matthias Grothe*, Soenke Langner, Felix von Podewils

*** Correspondence:** Corresponding Author: matthias.grothe@uni-greifswald.de

# Supplementary Tables

**Table 4** Significant clusters, their peak p-value and MNI coordinates of TBSS results (pPPR>nPPR, p<0.05 uncorrected). For the sake of brevity only the 25 largest clusters are shown.

| **Cluster size** | **p-value peak** | **X (mm)** | **Y (mm)** | **Z (mm)** |
| --- | --- | --- | --- | --- |
| 154 | <0.001 | -5 | 7 | 23 |
| 79 | 0.001 | 9 | 10 | 23 |
| 77 | 0.004 | 13 | 0 | 30 |
| 58 | <0.001 | 45 | -33 | -12 |
| 55 | 0.001 | -46 | -21 | -18 |
| 49 | 0.004 | 15 | -7 | 54 |
| 45 | <0.001 | 12 | -44 | 18 |
| 41 | 0.002 | 13 | -18 | 30 |
| 40 | 0.004 | -36 | 22 | 29 |
| 39 | <0.001 | -14 | 49 | 22 |
| 38 | 0.001 | 22 | -54 | 32 |
| 38 | 0.005 | -12 | -9 | -8 |
| 38 | 0.002 | -13 | 31 | 39 |
| 37 | 0.001 | -11 | -45 | 16 |
| 34 | 0.004 | 24 | -27 | -18 |
| 32 | 0.002 | -46 | -42 | 10 |
| 31 | 0.002 | -9 | 0 | 3 |
| 31 | 0.002 | -43 | -30 | 1 |
| 31 | 0.001 | 22 | -45 | 35 |
| 30 | 0.001 | -30 | 24 | -17 |
| 29 | 0.001 | 55 | 2 | 14 |
| 27 | 0.002 | 11 | -20 | -17 |
| 27 | <0.001 | -40 | -4 | -29 |
| 26 | 0.002 | -12 | 40 | 34 |
| 26 | 0.001 | -34 | 0 | -25 |

**
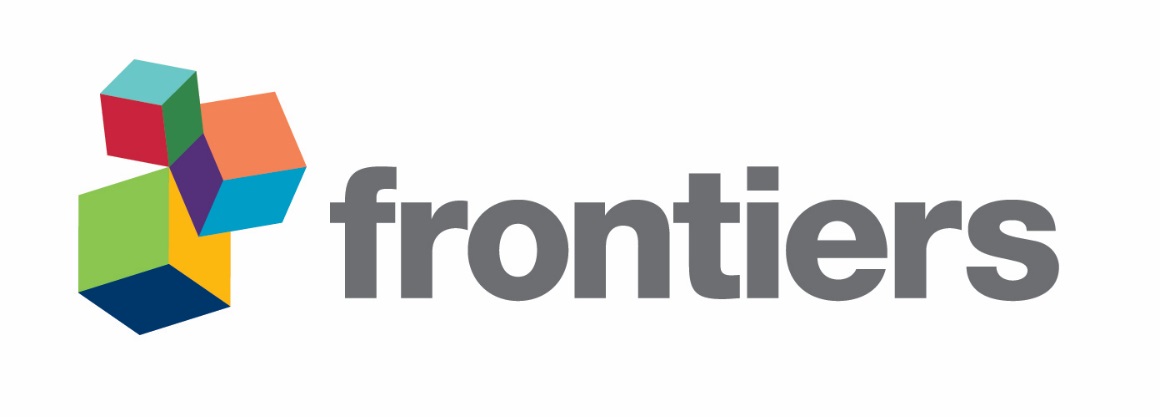
**
