# Supplementary material for: Juvenile Myoclonic Epilepsy Shows Potential Structural White Matter Abnormalities: A TBSS Study
Source: Front Neurol. 2018 Jun 29;9:509. doi: 10.3389/fneur.2018.00509 (PMC6033991; doi:10.3389/fneur.2018.00509)
Supplement: Supplementary file 3 [file Data_Sheet_3.docx]

Supplementary Material

Juvenile myoclonic epilepsy shows potential structural white matter abnormalities: a TBSS study

Martin Domin, Sabine Bartels, Julia Geithner, Zhong Irene Wang, Uwe Runge, Matthias Grothe*, Soenke Langner, Felix von Podewils

*** Correspondence:** Corresponding Author: matthias.grothe@uni-greifswald.de

# Supplementary Tables

**Table 5** Overlap of TBSS results (pPPR>nPPR, p<0.05 uncorrected) with “JHU ICBM-DTI-81 White-Matter Labels” and “JHU White-Matter Tractography Atlas”

| **JHU_ICBM-DTI-81_White-Matter_Labels** | **Overlap percentage** | **JHU_White-Matter_Tractography_Atlas** | **Average probability** |
| --- | --- | --- | --- |
| Body of corpus callosum | 42.4879 | Inferior longitudinal fasciculus L | 2.67398 |
| Splenium of corpus callosum | 7.53197 | Inferior longitudinal fasciculus R | 1.83117 |
| Sagittal stratum R | 2.76143 | Forceps minor | 1.40758 |
| Cingulum (hippocampus) R | 2.25694 | Anterior thalamic radiation L | 1.40532 |
| Cerebral peduncle L | 1.27451 | Cingulum (hippocampus) R | 1.01881 |
| Anterior limb of internal capsule L | 1.03554 | Forceps major | 0.939372 |
| Cerebral peduncle R | 0.920476 | Inferior fronto-occipital fasciculus R | 0.572687 |
| Sagittal stratum L | 0.902775 | Corticospinal tract R | 0.542152 |
| Posterior corona radiata R | 0.66823 | Superior longitudinal fasciculus R | 0.452228 |
| Posterior limb of internal capsule L | 0.632827 | Superior longitudinal fasciculus L | 0.446254 |
| Corticospinal tract R | 0.0708059 | Inferior fronto-occipital fasciculus L | 0.426207 |
|  |  | Superior longitudinal fasciculus (temporal part) R | 0.400053 |
|  |  | Superior longitudinal fasciculus (temporal part) L | 0.359561 |
|  |  | Cingulum (cingulate gyrus) L | 0.165995 |
|  |  | Uncinate fasciculus L | 0.0958092 |
|  |  | Anterior thalamic radiation R | 0.0939063 |
|  |  | Corticospinal tract L | 0.0533699 |
|  |  | Cingulum (hippocampus) L | 0.0148692 |
|  |  | Cingulum (cingulate gyrus) R | 0.0123468 |

**
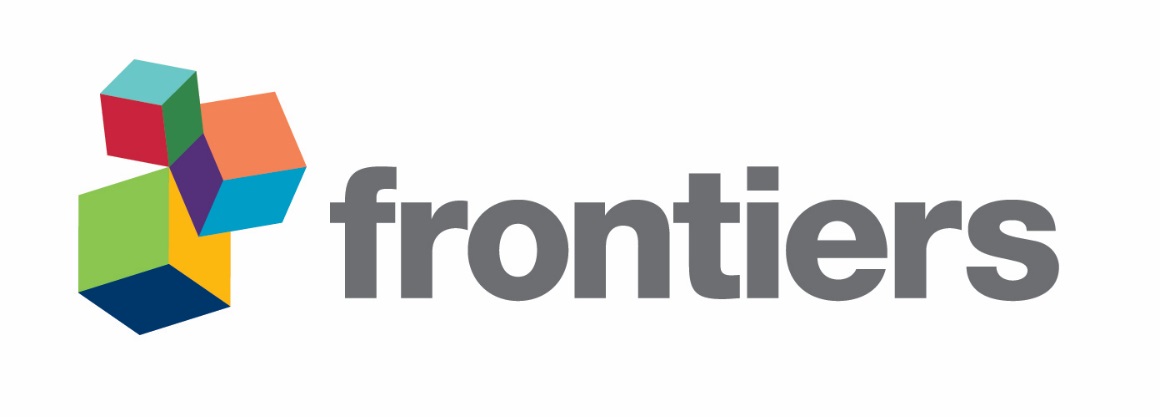
**
