# Supplementary material for: Juvenile Myoclonic Epilepsy Shows Potential Structural White Matter Abnormalities: A TBSS Study
Source: Front Neurol. 2018 Jun 29;9:509. doi: 10.3389/fneur.2018.00509 (PMC6033991; doi:10.3389/fneur.2018.00509)
Supplement: Supplementary file 4 [file Data_Sheet_4.docx]

Supplementary Material

Juvenile myoclonic epilepsy shows potential structural white matter abnormalities: a TBSS study

Martin Domin, Sabine Bartels, Julia Geithner, Zhong Irene Wang, Uwe Runge, Matthias Grothe*, Soenke Langner, Felix von Podewils

*** Correspondence:** Corresponding Author: matthias.grothe@uni-greifswald.de

# Supplementary Tables

**Table 6** Significant clusters, their peak p-value and MNI coordinates of TBSS results (pPPR<nPPR, p<0.05 uncorrected). For the sake of brevity only the 25 largest clusters are shown.

| **Cluster size** | **p-value peak** | **X (mm)** | **Y (mm)** | **Z (mm)** |
| --- | --- | --- | --- | --- |
| 157 | <0.001 | -14 | -85 | -35 |
| 85 | 0.001 | 15 | -89 | -32 |
| 76 | 0.002 | 23 | -81 | -36 |
| 53 | 0.001 | -25 | 9 | 28 |
| 49 | 0.001 | 34 | -10 | 38 |
| 46 | 0.002 | 27 | 40 | -5 |
| 41 | 0.004 | 2 | -26 | -28 |
| 41 | 0.001 | 24 | -54 | 43 |
| 40 | 0.003 | 33 | -22 | 36 |
| 37 | 0.002 | 17 | 15 | 28 |
| 37 | 0.001 | -20 | 52 | -7 |
| 35 | <0.001 | 36 | -77 | -29 |
| 32 | 0.003 | 23 | -43 | 48 |
| 32 | 0.002 | 19 | -34 | 60 |
| 31 | 0.002 | -25 | -2 | 34 |
| 31 | 0.007 | -16 | 6 | 45 |
| 29 | 0.004 | 11 | -47 | -55 |
| 29 | 0.002 | 39 | -19 | -30 |
| 28 | <0.001 | -17 | -54 | -52 |
| 27 | 0.001 | -26 | -90 | -6 |
| 27 | 0.003 | -21 | -82 | 11 |
| 26 | 0.001 | 28 | -80 | -26 |
| 25 | <0.001 | -43 | 4 | 14 |
| 25 | 0.005 | 17 | -32 | 9 |

**
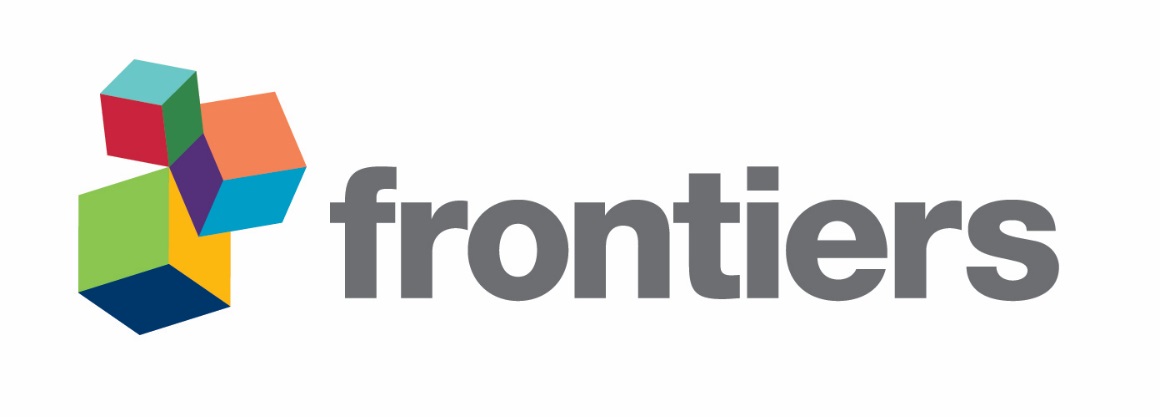
**
