# Supplementary material for: Juvenile Myoclonic Epilepsy Shows Potential Structural White Matter Abnormalities: A TBSS Study
Source: Front Neurol. 2018 Jun 29;9:509. doi: 10.3389/fneur.2018.00509 (PMC6033991; doi:10.3389/fneur.2018.00509)
Supplement: Supplementary file 5 [file Data_Sheet_5.docx]

Supplementary Material

Juvenile myoclonic epilepsy shows potential structural white matter abnormalities: a TBSS study

Martin Domin, Sabine Bartels, Julia Geithner, Zhong Irene Wang, Uwe Runge, Matthias Grothe*, Soenke Langner, Felix von Podewils

*** Correspondence:** Corresponding Author: matthias.grothe@uni-greifswald.de

# Supplementary Tables

**Table 7** Overlap of TBSS results (pPPR<nPPR, p<0.05 uncorrected) with “JHU ICBM-DTI-81 White-Matter Labels” and “JHU White-Matter Tractography Atlas”

| **JHU_ICBM-DTI-81_White-Matter_Labels** | **Overlap percentage** | **JHU_White-Matter_Tractography_Atlas** | **Average probability** |
| --- | --- | --- | --- |
| Superior corona radiata L | 7.70459 | Superior longitudinal fasciculus R | 3.88974 |
| Pontine crossing tract (a part of MCP) | 4.30966 | Superior longitudinal fasciculus (temporal part) R | 1.39131 |
| Superior longitudinal fasciculus R | 4.1746 | Corticospinal tract R | 0.511388 |
| Anterior corona radiata R | 1.47339 | Inferior fronto-occipital fasciculus R | 0.451102 |
| Body of corpus callosum | 0.920867 | Inferior fronto-occipital fasciculus L | 0.321812 |
| Superior corona radiata R | 0.736694 | Forceps major | 0.177911 |
| Anterior corona radiata L | 0.386764 | Anterior thalamic radiation L | 0.173369 |
|  |  | Superior longitudinal fasciculus L | 0.164344 |
|  |  | Uncinate fasciculus R | 0.136472 |
|  |  | Superior longitudinal fasciculus (temporal part) L | 0.135245 |
|  |  | Corticospinal tract L | 0.102769 |
|  |  | Anterior thalamic radiation R | 0.0742833 |
|  |  | Inferior longitudinal fasciculus R | 0.0672847 |
|  |  | Inferior longitudinal fasciculus L | 0.0589355 |
|  |  | Uncinate fasciculus L | 0.0438333 |
|  |  | Cingulum (cingulate gyrus) R | 0.0224692 |

**
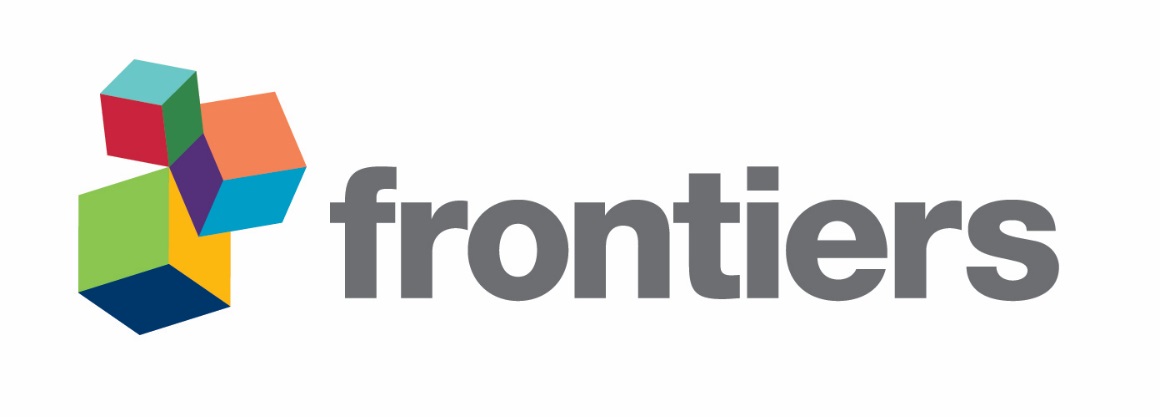
**
