# Supplementary material for: Juvenile Myoclonic Epilepsy Shows Potential Structural White Matter Abnormalities: A TBSS Study
Source: Front Neurol. 2018 Jun 29;9:509. doi: 10.3389/fneur.2018.00509 (PMC6033991; doi:10.3389/fneur.2018.00509)
Supplement: Supplementary file 6 [file Data_Sheet_6.docx]

Supplementary Material

Juvenile myoclonic epilepsy shows potential structural white matter abnormalities: a TBSS study

Martin Domin, Sabine Bartels, Julia Geithner, Zhong Irene Wang, Uwe Runge, Matthias Grothe*, Soenke Langner, Felix von Podewils

*** Correspondence:** Corresponding Author: matthias.grothe@uni-greifswald.de

# Supplementary Tables

**Table 8** Significant clusters, their peak p-value and MNI coordinates of TBSS results (correlation FA and duration of epilepsy, p<0.05 uncorrected). For the sake of brevity only the 25 largest clusters are shown.

| **Cluster size** | **p-value peak** | **X (mm)** | **Y (mm)** | **Z (mm)** |
| --- | --- | --- | --- | --- |
| 152 | <0.001 | -18 | 3 | 42 |
| 122 | <0.001 | -19 | -3 | 10 |
| 98 | 0.001 | -10 | 1 | 60 |
| 67 | <0.001 | 16 | 22 | 21 |
| 65 | 0.003 | -18 | -10 | -2 |
| 61 | <0.001 | 27 | 21 | 18 |
| 59 | <0.001 | 54 | -34 | 14 |
| 58 | 0.001 | 30 | -20 | 44 |
| 57 | 0.001 | 25 | 34 | 20 |
| 56 | 0.001 | 23 | -32 | 50 |
| 55 | 0.001 | 47 | -16 | -3 |
| 54 | <0.001 | -41 | -36 | 2 |
| 53 | 0.001 | -20 | -20 | -10 |
| 52 | 0.001 | -26 | -56 | 34 |
| 52 | <0.001 | 39 | -42 | 18 |
| 51 | 0.002 | -26 | -69 | 12 |
| 51 | 0.005 | 20 | -16 | 45 |
| 49 | 0.001 | 26 | 25 | 21 |
| 48 | 0.001 | -43 | 1 | -24 |
| 47 | 0.002 | -33 | 20 | 31 |
| 44 | <0.001 | -26 | -42 | 38 |
| 43 | 0.005 | -36 | -46 | 26 |
| 41 | <0.001 | -28 | -22 | 44 |
| 40 | <0.001 | 31 | -41 | 32 |
| 39 | <0.001 | -10 | -14 | -15 |

**
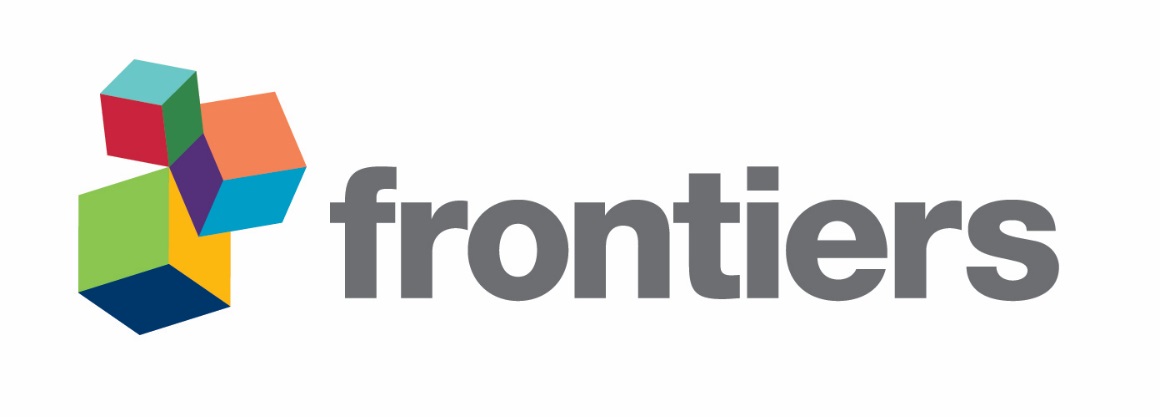
**
