# Supplementary material for: Juvenile Myoclonic Epilepsy Shows Potential Structural White Matter Abnormalities: A TBSS Study
Source: Front Neurol. 2018 Jun 29;9:509. doi: 10.3389/fneur.2018.00509 (PMC6033991; doi:10.3389/fneur.2018.00509)
Supplement: Supplementary file 7 [file Data_Sheet_7.docx]

Supplementary Material

Juvenile myoclonic epilepsy shows potential structural white matter abnormalities: a TBSS study

Martin Domin, Sabine Bartels, Julia Geithner, Zhong Irene Wang, Uwe Runge, Matthias Grothe*, Soenke Langner, Felix von Podewils

*** Correspondence:** Corresponding Author: matthias.grothe@uni-greifswald.de

# Supplementary Tables

**Table 9** Overlap of TBSS results (correlation FA and duration of epilepsy, p<0.05 uncorrected) with “JHU ICBM-DTI-81 White-Matter Labels” and “JHU White-Matter Tractography Atlas”

| **JHU ICBM-DTI-81 White-Matter Labels** | **Overlap percentage** | **JHU White-Matter Tractography Atlas** | **Average probability** |
| --- | --- | --- | --- |
| Posterior limb of internal capsule L | 6.9431 | Superior longitudinal fasciculus R | 2.15929 |
| Anterior corona radiata R | 5.05392 | Inferior longitudinal fasciculus L | 2.09747 |
| Cerebral peduncle L | 4.58535 | Corticospinal tract L | 1.81951 |
| Genu of corpus callosum | 3.66586 | Superior longitudinal fasciculus L | 1.6707 |
| Superior longitudinal fasciculus R | 3.39438 | Anterior thalamic radiation L | 1.47039 |
| Anterior limb of internal capsule L | 2.57717 | Inferior fronto-occipital fasciculus L | 1.12024 |
| Superior longitudinal fasciculus L | 1.38062 | Superior longitudinal fasciculus (temporal part) R | 1.07064 |
| Cingulum (hippocampus) L | 1.30718 | Corticospinal tract R | 0.819199 |
| Superior corona radiata R | 1.29416 | Superior longitudinal fasciculus (temporal part) L | 0.706685 |
| Superior corona radiata L | 1.24954 | Forceps major | 0.683265 |
| Sagittal stratum L | 1.16586 | Forceps minor | 0.658693 |
| Cingulum (hippocampus) R | 1.0543 | Inferior fronto-occipital fasciculus R | 0.532596 |
| Retrolenticular part of internal capsule L | 0.542023 | Anterior thalamic radiation R | 0.46852 |
| Posterior thalamic radiation (include optic radiation) L | 0.509483 | Cingulum (hippocampus) R | 0.420463 |
| Anterior limb of internal capsule R | 0.442544 | Cingulum (hippocampus) L | 0.352826 |
| Middle cerebellar peduncle | 0.290071 | Uncinate fasciculus L | 0.322713 |
| Body of corpus callosum | 0.238936 | Inferior longitudinal fasciculus R | 0.301144 |
| Tapetum L | 0.214764 | Cingulum (cingulate gyrus) L | 0.0491447 |
| External capsule L | 0.13016 | Cingulum (cingulate gyrus) R | 0.023875 |
|  |  | Uncinate fasciculus R | 0.0084511 |

**
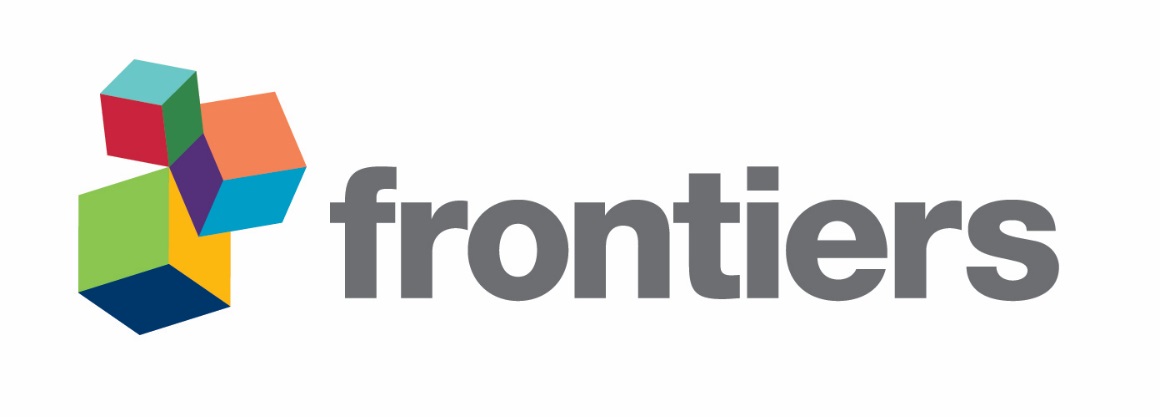
**
